# Trading Community Analysis of Countries’ Roll-On/Roll-Off Shipping Networks Using Fine-Grained Vessel Trajectory Data

**DOI:** 10.3390/s24227226

**Published:** 2024-11-12

**Authors:** Shichen Huang, Tengda Sun, Jing Shi, Piqiang Gong, Xue Yang, Jun Zheng, Huanshuai Zhuang, Qi Ouyang

**Affiliations:** China Transport Telecommunications and Information Center, Beijing 100011, China; 15210576313@163.com (S.H.);

**Keywords:** roll-on/roll-off vessels, vessel trajectory data, complex network, community detection, graph convolutional neural network

## Abstract

Roll-on/roll-off vessels (RO/RO vessels) are playing an increasingly critical role in international automobile transport, facilitating the efficient movement of vehicles and heavy machinery across continents. Despite this growing significance, there is still limited research specifically focused on the RO/RO shipping network and its impact on global trade. This paper studies the global RO/RO shipping network using AIS data on RO/RO vessels collected from 2020 to 2023. We construct a method based on the complex network theory and the graph feature extraction method to quantitatively assess the features of the RO/RO shipping network. This method assesses the complexity, sparsity, homogeneity, modularity, and hierarchy of the RO/RO shipping network across various ports and countries and employs the graph convolutional neural network (GCN) model to extract network features for community detection. This process enables the identification of port clusters that are frequently linked to RO/RO vessels, as well as regional transport modes. The paper’s findings support these conclusions: (1) From 2020 to 2023, the number of nodes in the RO/RO shipping network increased by 22%, primarily concentrated in African countries. The RO/RO shipping network underwent restructuring after the pandemic, with major complex network parameters showing an upward trend. (2) The RO/RO shipping network is complex, with a stable graph density of 0.106 from 2020 to 2023. The average degree increased by 7% to 4.224. Modularity decreased by 6.5% from 0.431 in 2022 to 0.403, while the hierarchy coefficient rose to 0.575, suggesting that post-pandemic, community routes have become more diverse, reflecting the reconstruction and maturation of the overall network. (3) The model yielded a silhouette coefficient of 0.548 and a Davies–Bouldin index of 0.559 using an improved automatic feature extraction method. In comparison between 2020 and 2023, the changes in the two indicators are small. This shows that GINs can effectively extract network features and give us results that we can understand for community detection. (4) In 2023, key communities divide the RO/RO shipping network, with one community handling 39% of global routes (primarily Europe–Asia), another community handling 23% (serving Asia–Pacific, Africa, and the Middle East), and a third community managing 38% (linking Asia, Europe, and South America).

## 1. Introduction

RO/RO vessels, known for their efficiency, versatility, and cost-effectiveness, play a crucial role in the flow of goods between countries [1,2]. These vessels are particularly competitive for high-value and time-sensitive cargo, offering significant reductions in total logistics costs [3]. In recent years, the demand for RO/RO vessels has surged, with major shipping companies such as EPS, Smyril Line, Grimaldi Group, and Toyofuji Shipping placing new orders for RO/RO vessel construction. This indicates that global RO/RO shipping activity is likely to continue growing soon.

The global pandemic has had a direct impact on the global economy [4,5]. The container shipping network [6], as well as tanker and bulk shipping [7], faced significant operational challenges, with the RO/RO shipping routes also being notably impacted [8]. As the pandemic was gradually brought under control, the international shipping market showed signs of recovery amidst volatility, with RO/RO transport operations resuming incrementally. In May 2023, the United Nations [9] declared that the pandemic no longer constituted a Public Health Emergency of International Concern (PHEIC).

Studying the RO/RO shipping network between nations and the reconstruction process of the RO/RO shipping network under the influence of the pandemic will enhance our understanding of the global automotive trade pattern. Furthermore, such research provides scientific support for countries involved in the automotive trade to adjust and optimize their production policies, ensuring the automotive sector’s sustainable development.

Studies conducted in the past five years on RO/RO shipping mainly focus on energy technologies [10], emissions [11], efficiency optimization [12], and transport safety [13], but rarely touch on the global RO/RO shipping network. That is, despite the rich research results obtained in the above fields, there is a lack of research on the global RO/RO shipping network. Shipping networks are an important field for applying graph theory in practice. Many studies have investigated the shipping networks of different regions in the world, such as Asia and Europe, and explored the network features of different vessel types, like container vessels, liquefied natural gas (LNG) carriers, etc. They have also investigated the impact of COVID-19 on shipping networks, as well as the impact of major incidents (such as the Suez Canal blockage) on the global shipping network. However, there are relatively few studies dedicated to the RO/RO shipping network.

This paper proposes a novel method to quantitatively assess the features of the RO/RO shipping network, combining the complex network theory with the graph feature extraction technique. By analyzing the complexity, sparsity, homogeneity, modularity, and hierarchy of the RO/RO shipping network, this method can assess the performance of different ports and countries in the global RO/RO shipping network. Additionally, we use a GCN model to extract key network features, perform community detection, and identify the major port clusters frequented by RO/RO vessels, along with their regional transport modes.

The findings of this paper offer scientific guidance for automobile manufacturers and logistics enterprises in formulating their global layout and operational strategies, helping them optimize the global automobile logistics network, reduce costs, and improve overall operational efficiency. This paper also provides theoretical assistance to governments and relevant policymakers in conducting port construction and formulating trade policies, contributing to the healthy development of the global automobile trade. This paper aims to put forward insightful views on the future development of the global RO/RO shipping network, based on in-depth analysis and discussions, and to offer references for the optimization of the international logistics system in the course of global economic integration.

## 2. Literature Review

### 2.1. Analysis of Shipping Networks Based on the Complex Network Theory

The growth of global trade is constantly increasing the complexity and importance of shipping networks. The complex network theory, as a powerful tool, has been widely used in research on shipping networks to reveal their structural features, assess their vulnerability, optimize shipping route layout, etc. The progress of studies conducted in recent years on the analysis of shipping networks using the complex network theory is summarized below.

In terms of key port identification and network robustness, Guerrero [14] applied complex network principles to analyze the impact of the COVID-19 pandemic on the global maritime container shipping network and related ports. Qian [15] used complex network methods to analyze the topological characteristics of the Southeast Asian shipping network, identifying hub ports and evaluating the network’s robustness through attack simulations. Peng [16] analyzes the global distribution trends of container traffic across more than 1000 ports from 2013 to 2022 to assess the dominance and influence of ports. Peng [17] developed a comprehensive CCPE evaluation model to assess port competitiveness using 18 factors related to conditions, capacity, potential, and efficiency. This model leverages big data on the geographical environment, cargo vessel trajectories, port infrastructure, and regional socioeconomic factors.

In terms of the structural features and evolutionary trends of shipping networks, Wang [18] reviewed methods for evaluating port dominance, emphasizing that with the advancement of information technology, evaluation techniques are evolving towards more comprehensive and intelligent approaches. Peng [19,20] utilized a series of complex network metrics and a community detection approach to examine global LPG and LNG trade patterns and their evolution, focusing on trading communities at the port level, based on extensive vessel trajectory data gathered from 2013 to 2017. Zhang [21] utilized complex network methods to analyze the structural characteristics and evolution of the US container shipping network during a specific period. The study also highlighted the impact of COVID-19, and proposed recommendations for the development of hub ports and the balanced growth of the network. Ge [22] introduced the concept of motif detection, identifying connection patterns among three to four ports in liner shipping services. They proposed two regional connection patterns: branch and circular. Ansorena [23] identified the community structure of the global shipping network through modularity optimization and analyzed the strongest communities using centrality measures, revealing the high-performing countries over the past decade. Calatayud [24] revealed the vulnerability of international freight flows in multi-layered liner shipping networks through modeling and simulation analysis, and noted that the extent of disruption depends on a country’s role within the network. Wu [25], by detecting port communities in the global liner shipping network, analyzed the spatial heterogeneity of port communities with different sizes, and revealed the structural features of port communities under the regionalization trend and their effects on maritime trade. Zhang [26] analyzed the statistical properties of the Maritime Silk Road using complex network analysis and found that its heavy reliance on hubs in the shipping network may lead to vulnerabilities.

In terms of shipping network optimization and assessment frameworks, Li [27] explored the impact of COVID-19 on the flow and vulnerability of the network and the positions of its major hub ports using the complex network theory. Xu [28] developed a comprehensive framework to assess the efficiency and vulnerability of the global liner shipping network. Wang [29] adopted the complex network method to analyze the spatial pattern of shipping networks before and after the merger and reorganization of China Ocean Shipping (Group) Company (COSCO) and China Shipping Container Lines Co., Ltd., into China COSCO Shipping Corporation Limited (COSCO Shipping), and assessed its integration effects in terms of network economy and hub economy. Peng [30] presents a versatile multi-layer network approach for analyzing an international maritime network, highlighting its ability to effectively identify various transportation flows and adaptable ports within the system.

### 2.2. Graph Convolutional Neural Networks (GCNs) for Feature Extraction

Graph data are a common form of data in both natural and artificial systems, as many of the patterns we observe can be elegantly represented by graph structures. Typical examples in this respect include molecular structures (graphs composed of atoms and chemical bonds), social networks, traffic networks, etc. The powerful potential of such graph representations has been recognized by scientific research and industrial applications in fields as diverse as traffic flow prediction, drug discovery, social network analysis, and personalized recommendation systems. Kipf [31] designed the graph convolutional network (GCN), which considers the neighbor relationships in the graph structure to represent the interactions between nodes. It aggregates the features of neighboring nodes to generate the feature representation of the target node. Inspired by the graph isomorphism test, Xu [32] proposed the GIN model, which significantly improves the representational power of graph neural networks. Velickovic [33] introduced an attention mechanism similar to that of transformers and designed the GAT model, where the attention weights between nodes and their neighbors are calculated during the node weight update process.

In terms of road traffic, Wen [34] proposed Graph2Route, a pickup/delivery route prediction model based on dynamic spatio-temporal graphs. Diao [35] introduced CRFAST-GCN, a multi-branch spatio-temporal attention graph convolutional neural network that can effectively process complex spatio-temporal dynamics, capture multi-scale dependencies and semantic similarities in traffic data, and complete traffic flow prediction tasks. Wang [36] presented a novel spatio-temporal graph neural network (STGNN) model for traffic flow prediction, which effectively captured spatio-temporal dependencies through a location attention mechanism and sequence components. Gammelli [37] raised a deep reinforcement learning framework based on graph neural networks (GNNs) to control the rebalancing of automated mobility-on-demand systems.

In terms of water traffic, Patel [38] developed a novel deep learning method combining GNNs and the You Only Look Once v7 (YOLOv7) deep learning framework, which enhanced the ability to understand the presence of vessels in port areas. Altan [39] put forward a port classification method based on temporal graph neural networks (TGNNs) for the efficient identification of gateway ports and their relevant features. Xin [40] came up with a GNN-based semi-supervised deep learning method to identify vessel movement behavior patterns based on AIS data. Li [41] brought forward a semi-dynamic spatio-temporal graph neural network (SDSTGNN) for traffic state prediction in waterways. Luo [42] proposed AISim, a learning framework for AIS trajectory measurement based on heterogeneous GNNs. Li [43] presented an STGNN model combining graph attention networks (GATs) and the dilated causal convolution architecture for vessel flow prediction in multi-port scenarios. Zheng [44] proposed a new model called VDGCNeT, which combines the advantages of graph convolutional networks and transformers. Tests on two real-world traffic datasets demonstrated that this model significantly outperforms other models in traffic flow prediction. Saadat [45] introduced a model that combines CNN, ALO, and SOM for automatically extracting features and accurately classifying network traffic types, including identifying encrypted traffic and distinguishing VPN traffic. This model achieved 98% accuracy on the ISCX dataset. Gianni [46] proposed a deep learning architecture combining autoencoders, convolutional neural networks, and recurrent neural networks for the automated extraction of high-level features in network traffic classification. Experiments showed significant improvements in accuracy, and the method performed well even on imbalanced datasets.

Although the RO/RO shipping network plays an irreplaceable role in global automobile trade, existing studies in this field are still insufficient in many aspects. As far as existing network analysis is concerned, there is often a lack of in-depth quantification of network features such as complexity, sparsity, homogeneity, modularity, hierarchy, and symmetry, which leads to an incomplete understanding of network features. An algorithm is required to automatically extract features of the RO/RO shipping network at the country level.

## 3. Data and Methodology

### 3.1. Data and Network Construction

**Figure 1:**
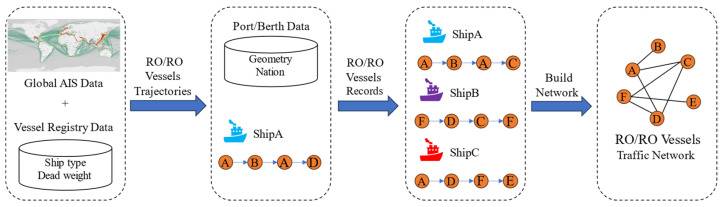
Network constructed diagram based on AIS.

This study focuses on the RO/RO vessels relations between nations. Hence, as shown in Figure 1, the global AIS data and vessel registry information, such as ship type and deadweight tonnage, are first used to track the navigational trajectories of RO/RO vessels. Next, these trajectories are matched with national and berth data to generate entry and exit records for vessels in different countries. These records depict the actual routes traveled by each ship between nations, forming the OD (origin–destination) data. A shipping network with ports as nodes and RO/RO vessels’ navigation paths as edges is established. *G = {V,E}*, where *V = {“all nations to which RO/RO vessels navigate”}* and *E = {“nation pairs”}*. When establishing a shipping network, the numbers of navigations by different RO/RO vessels between each of these port pairs are adopted as weights.

For a comprehensive study of data on the RO/RO shipping network over a long period of time, AIS data were collected for 858 RO/RO vessels with a deadweight tonnage (DWT) of ≥10,000 t, which had participated in international voyages around the world. These vessels were collected from Lloyd’s Register of Shipping, and the AIS data, covering the period from 2020 to 2023, were processed to identify vessel entry and exit events between countries. From 2020 to 2023, the average growth rate of records was 7.1%, and a total of 33,470 records were obtained in 2023. Similarly, both the number of routes and the number of nations increased, as detailed in Table 1.

### 3.2. Methodology

**Figure 2:**
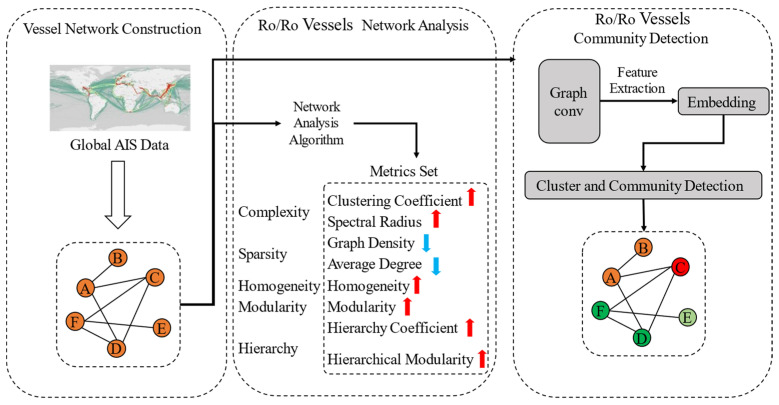
Methodology flowchart.

Taking RO/RO vessels as the research object, this paper utilizes AIS data to conduct an in-depth analysis of RO/RO vessel network characteristics using advanced techniques such as complex network analysis, graph convolutional networks (GCNs), and clustering analysis. The study examines five key dimensions. By extracting structural features from the graph, community detection is performed to reveal potential clusters and shipping patterns within the network. Additionally, the paper integrates China’s rapid growth in automobile exports and its overseas factory expansion strategy to comparatively analyze the changes in network characteristics under different influencing factors. It explores the impact on global trade flows, port connectivity, and the structure of the shipping network as they relate to future developments. Ultimately, this paper provides a robust theoretical foundation for understanding the role of RO/RO vessel shipping networks in global supply chains and offers valuable insights for policymakers and industry stakeholders in optimizing shipping routes and port layouts, as shown in Figure 2.

#### 3.2.1. Analysis of Network Features

Many complex systems, including transport networks, can be abstracted as network structures. By analyzing the features of a network structure, we can gain an in-depth understanding of its operational mechanisms and characteristics. For a systematic analysis on the features of the RO/RO shipping network, basic indicators such as simple dependency can provide preliminary information, but they are often insufficient to fully reveal complex network structures or their potential behaviors. In view of this, this paper analyzes network structures from five dimensions, namely, complexity, sparsity, homogeneity, modularity, and hierarchy.

Complexity uncovers the complex interactions between nodes and edges in the network. Sparsity reflects the density of the network. Homogeneity describes the similarity in attributes between nodes and edges in the network. Modularity exposes potential community structures in the network. Hierarchy exhibits the importance and functional layering of nodes in the network.

As shown in Table 2, complexity is measured by the average clustering coefficient, spectral entropy, and spectral radius [47,48]. Sparsity is measured by the graph density, average degree, and degree distribution [47]. Homogeneity is measured by the structural homogeneity [49]. Modularity is measured by the degree of modularity. Hierarchy is measured by the hierarchical coefficient, hierarchical modularity, and hierarchical entropy [50].

#### 3.2.2. GNN-Based Feature Extraction and Community Detection

When it comes to the feature extraction of the RO/RO shipping network, there has been no systematic study that compares the effects of different feature extraction models. In this sense, exploring and assessing the applications of various automatic graph feature extraction models in the field of the RO/RO shipping network will not only fill in the gaps in existing studies but also offer important references for future research.

(1) Feature extraction model design

**Figure 3:**
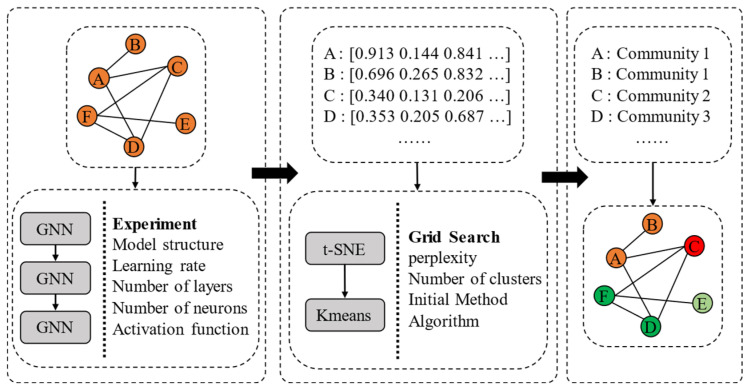
Methodology flowchart for GNN-based feature extraction and community detection.

In the comparison and design of feature extraction models, GCNs, GINs, and GATs are selected in this paper based on Figure 3. GCNs can be used to realize the convolutional aggregation of node features via the graph Laplace operator. GINs are particularly applicable to scenarios with complex and diverse network structures. GATs, by introducing an attention mechanism, allow the model to dynamically adjust the weights of neighbor nodes. In terms of parameter setting, to enable all models to fully capture the structural features of the network and endow them with a sound generalization ability, this paper optimizes the key parameters of each model.

(2) Dimensionality reduction-based clustering and community detection

As shown in Figure 3, this paper introduces a method that combines t-distributed stochastic neighbor embedding (t-SNE) dimensionality reduction and clustering analysis. High-dimensional features extracted by GNNs are first mapped to a 2D space using t-SNE, allowing the degree of clustering among nodes representing different countries to be visualized while preserving essential node characteristics. Following dimensionality reduction, the K-means clustering algorithm is applied to identify potential communities within the network. Each cluster generated by K-means represents a potential community, revealing groups of tightly connected nodes within the RO/RO shipping networks. The number of clusters and other K-means parameters are selected to maximize clustering accuracy and enhance community clarity.

Performance optimization is achieved through hyperparameter tuning via grid search. The t-SNE algorithm’s perplexity parameter is optimized to balance local and global structures during embedding. For K-means, the optimal number of clusters, initialization method, and optimization algorithm are identified. This grid search process ensures that the most representative clustering configuration for the dataset is obtained.

(3) Evaluation indicators

This paper evaluates the extraction results using visual and quantitative indicators of dimensionality reduction. The silhouette coefficient and Davies–Bouldin index are used as indicators. These indicators aim to provide guidance for the optimization of model parameters.

## 4. Results

### 4.1. Network Feature Analysis

#### 4.1.1. Node Degree

**Figure 4:**
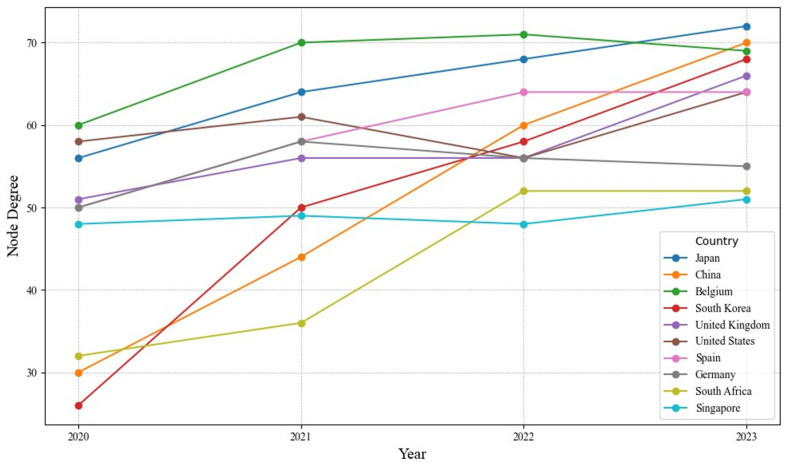
The top ten countries of the node degree of country-level networks.

The node degrees of country-level RO/RO shipping networks (hereinafter referred to as “country-level networks”) are calculated, and the top ten countries in terms of node degree are selected, as shown in Figure 4.

As shown in Figure 4, from 2020 to 2023, the RO/RO shipping network node degree of each country generally showed an upward trend, reflecting the gradual recovery and reconfiguration of the global RO/RO shipping network following the pandemic. The data trend can be divided into two phases. In the first phase (2020–2022), the RO/RO shipping network exhibited a trend toward intensification, particularly in South Korea (increasing from 26 to 50) and China (from 30 to 44), indicating that, in the early pandemic period, countries actively adjusted port connection structures to cope with supply chain pressures. In the second phase (2022–2023), the node degree of the RO/RO shipping network continued to grow. In Europe, the node degrees remained relatively stable, as seen in countries like the United Kingdom, Belgium, and Germany, highlighting adaptability and resilience within the RO/RO network. In Asia, node degrees continued to increase, particularly for Japan (reaching 72) and China (growing to 70), underscoring the solid and rising position of these regions within the RO/RO shipping network.

#### 4.1.2. Clustering Coefficient

From 2020 to 2023, the clustering coefficient of the RO/RO shipping network gradually increased from 0.56 to 0.63, indicating a higher probability of closed triangular structures between nodes within the network. This increase in the clustering coefficient suggests a rise in direct shipping routes between countries, strengthening network connectivity and integrating more countries into interconnected clusters. The slight decline in the clustering coefficient in 2022 reflects adjustments in the RO/RO shipping layout between countries during the pandemic, as detailed in Table 3. In 2023, the clustering coefficient rose to 0.63, which indicates the high proximity between countries in the global shipping network. In other words, there were direct shipping connections between neighboring countries. This also suggests that the network had strong modular features, and that these countries formed a close sub-network or regional shipping hub.

#### 4.1.3. Node Degree Centrality

**Figure 5:**
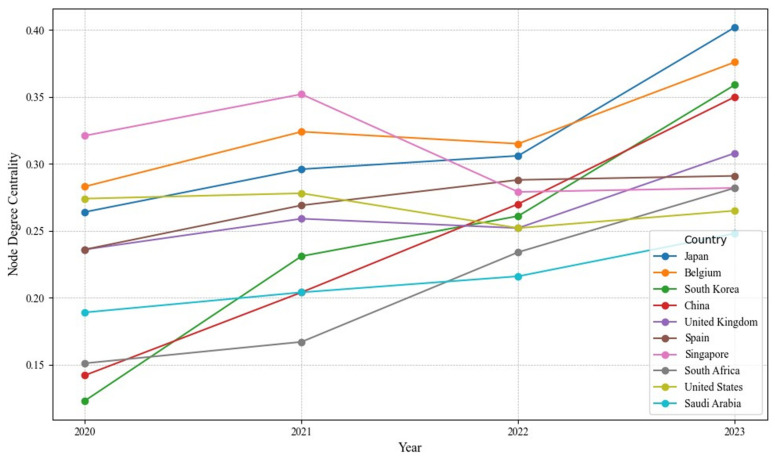
The top countries of node degree centrality of country-level networks.

As shown in Figure 5, from 2020 to 2023 the node degree centrality within the global RO/RO shipping network showed an overall upward trend, reflecting the recovery of supply chains post-pandemic and the growing demand for enhanced connectivity at key ports. Countries like Japan, Belgium, and South Korea experienced significant increases in node degree centrality, further solidifying their positions. In particular, Belgium and Japan, located in Europe and Asia, respectively, reached centrality scores of 0.376 and 0.402 in 2023, highlighting their robust connectivity. In contrast, traditional hubs like the United States and Spain showed more moderate growth, suggesting that their relative importance may be impacted by competition from emerging markets and network restructuring.

Japan’s node degree centrality rose from 0.264 in 2020 to 0.402 in 2023, indicating an increasing role for Japanese ports in the global transport of automobiles and heavy equipment. Ports like Yokohama and Nagoya recovered swiftly after the pandemic and actively expanded routes to Asia and North America. Similarly, South Korea’s node degree centrality increased from 0.123 to 0.359, underscoring the strategic importance of Busan and Pyeongtaek ports for Asian trade and transshipment. Belgium’s node degree centrality rose from 0.283 to 0.376, driven by Antwerp’s role as a major European hub, achieving a level of global connectivity surpassing even larger economies. This highlights the impact of regional hub status and advanced infrastructure on a country’s centrality within the network. South Africa’s node degree centrality grew from 0.151 to 0.282, underscoring its bridge role between Africa and the global shipping network, gradually establishing it as a strategic logistics node in the Southern Hemisphere.

#### 4.1.4. Hub Nations

**Figure 6:**
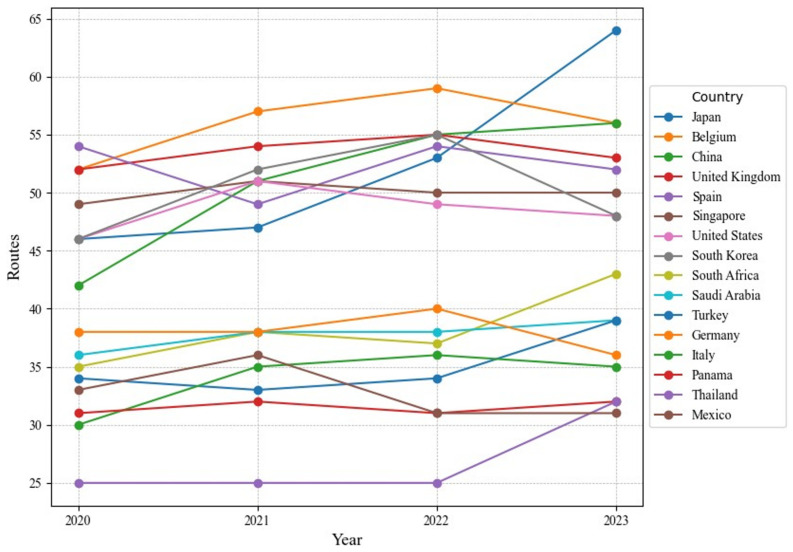
The top 16 countries of routes in country-level networks.

From an overall perspective, the number of routes in the global RO/RO shipping network exhibited varied fluctuations from 2020 to 2023, with adjustments seen across nations, particularly in major logistics hubs and emerging markets. Some countries, such as Japan and China, gradually increased their route numbers, underscoring their growing importance in the reconfiguration of the global RO/RO supply chain. In contrast, traditional logistics powerhouses like the United States and Germany maintained relatively stable route counts, as detailed in Figure 6.

In Asia, Japan’s RO/RO route count increased from 46 in 2020 to 64 in 2023. Under the Belt and Road Initiative, China’s influence in Asia and Africa expanded significantly, with its route count rising from 42 in 2020 to 56 in 2023. Singapore maintained a stable route count, reflecting its mature position within the global RO/RO shipping network.

In Europe, Belgium, the United Kingdom, Germany, and Italy had average route counts of 56, 53.5, 38, and 34, respectively, from 2020 to 2023, with minimal fluctuations. This stability indicates a highly consistent RO/RO market in Europe. Compared to the growth in the Asian RO/RO routes, the United States’ route count remained steady, with an average of 48.5, reflecting a stable trend in its RO/RO connections.

**Figure 7:**
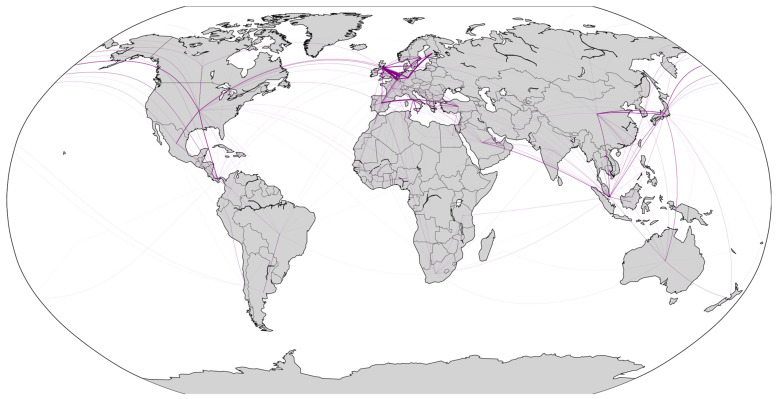
Global RO/RO shipping networks with weighted connections (2020).

**Figure 8:**
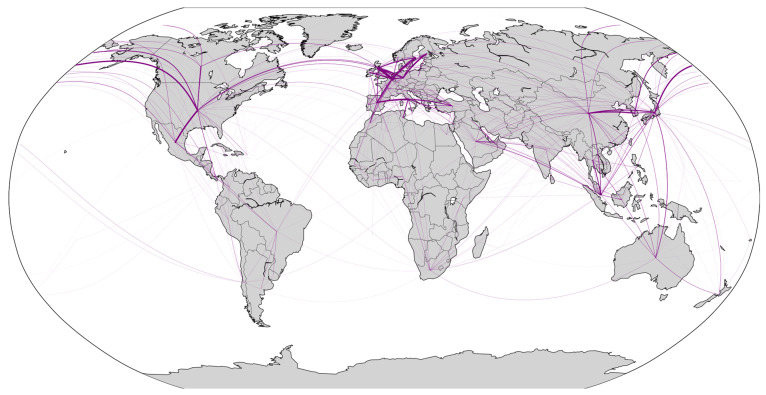
Global RO/RO shipping networks with weighted connections (2023).

As shown in Figure 7 and Figure 8, the geographic spatial characteristics and regional distribution of the network reveal a global reach, with key regions centered in Europe, North America, and East Asia, primarily concentrated in the Northern Hemisphere. Within Europe and among the three major East Asian countries, a dense network is observed, reflecting the port clustering effect in these regions. In the Southern Hemisphere, major countries such as South Africa, Australia, Brazil, and Argentina are connected by RO/RO shipping routes. The RO/RO shipping network displays a cross-regional pattern, with dense routes linking East Asia, Europe, and North America, indicating a busy trade network for RO/RO shipments between these regions. It is evident that the global RO/RO shipping network includes intra-community routes and inter-community connections.

Comparing the global RO/RO shipping route maps from 2020 and 2023, we can observe the network’s recovery and reconfiguration in the post-pandemic period. Compared to 2020, the 2023 map shows increased density within Europe and East Asia, especially with more connections between Northern European ports. This trend indicates that, post-pandemic, ports within these regions strengthened regional links to enhance supply chain resilience. Additionally, North American routes in 2023 expanded compared to 2020.

The 2023 RO/RO network exhibits both deepened globalization and regionalization trends compared to 2020. Globalization is reflected in the increased cross-regional connections from Asia to Europe and from North America to South America. Regionalization, on the other hand, is evident in the dense short-distance routes within Europe, East Asia, and Southeast Asia. This dual trend reflects the post-pandemic supply chain’s dual need to meet global demands while ensuring regional stability.

Overall, the RO/RO shipping network for vehicle transport reveals each country’s unique position and dependency within the global automotive supply chain. Whether it be manufacturing powerhouses like Japan and China, or strategic hubs like Belgium and Panama, their RO/RO shipping network underpins the smooth flow of global automotive trade.

#### 4.1.5. Comprehensive Evaluation Indicators

As can be seen from Table 4, this paper comparatively analyzes the structural features of country-level networks and random graphs of the same scale. By analyzing the five dimensions of complexity, sparsity, homogeneity, modularity, and hierarchy, we reveal the significant structural differences between the RO/RO shipping network and random graphs.

From 2020 to 2023, various complex network metrics of the global RO/RO shipping network reveal distinct structural characteristics and dynamic changes.

The average clustering coefficient increased from 0.561 in 2020 to 0.656 in 2023, indicating an enhancement in network clustering, with more direct connections between countries, which strengthens network connectivity and resilience. Compared to a random network, the clustering coefficient of the network is significantly higher, suggesting a highly concentrated cluster structure in terms of geography and function. Graph entropy rose gradually from 4.244 in 2020 to 4.538 in 2023, reflecting an increase in network information complexity, improved node connectivity uniformity, and greater network robustness. Similarly, the average degree grew from 22.43 in 2020 to 28.64 in 2023, showing that the average number of connections for each country within the network increased. This indicates that more ports participated in the global network, enhancing network accessibility. Graph density remained relatively stable over these four years, suggesting a tendency towards stable overall connectivity within the network. Changes in average clustering coefficient, graph entropy, average degree, and graph density over these four years demonstrate a densified and enriched global RO/RO network with improved robustness.

The spectral radius showed an upward trend from 2020, peaking in 2022 before decreasing in 2023. This decline in spectral radius reflects a redistribution of shipping routes. In contrast, the spectral radius of a random network was lower, indicating the high connectivity and complexity of the network. Structural homogeneity returned to its 2020 level in 2023, after a decline in 2021 and 2022. This trend reflects port structure fluctuations and adjustments during the pandemic. However, the recovery in structural homogeneity in 2023 suggests a return to uniform connectivity, as clustering intensified within the network. Modularity decreased from 0.431 in 2020 to 0.403 in 2023, indicating that during the pandemic, routes were more concentrated within communities, while post-pandemic, the network became more integrated overall. Changes in spectral radius, structural homogeneity, and modularity reflect the fluctuations and restructuring of the network under pandemic conditions.

The hierarchy coefficient increased from 0.44 in 2020 to 0.575 in 2023, and hierarchical modularity reached 1.087 in 2023. These metrics indicate a more pronounced hierarchical structure and modular characteristics within the network, with ports exhibiting a tiered distribution. Compared to a random network, the hierarchical nature of the network was more prominent, showing that in the post-pandemic supply chain restructuring, countries such as China, Singapore, and the United States gradually evolved into regional hubs, taking on greater distribution and connectivity roles.

From 2020 to 2023, the complexity and clustering characteristics of the global RO/RO shipping network progressively intensified, showing a trend of multipolarity and clustering. As the supply chain restructured post-pandemic, the centrality of key ports increased, and a hierarchical structure gradually formed. Compared to a random network, the global RO/RO shipping network’s connectivity and structural properties exhibited a higher degree of organization.

### 4.2. Feature Extraction and Community Detection Analysis of the RO/RO Shipping Network

#### 4.2.1. Experimental Results

In the stage of feature extraction model design, hyperparameters are optimized mainly from the aspects of model structure, learning rate, activation function, parameter normalization, optimizer, etc.

Compared with the models, we found a better set of hyperparameters, where the base model is GIN, the number of layers is three, the number of neurons in each layer is eight, the activation function is ReLU, and the learning rate is 5 × 10^−5^. In 2020, the model results classified countries into eight clusters, with the proportion of the largest number of countries in a single community being 22.7%. The silhouette coefficient was 0.570, and the Davies–Bouldin index was 0.534. In 2023, the results showed eight clusters, with the proportion of the largest number of countries in a single community being 21.1%, the silhouette coefficient being 0.548, and the Davies–Bouldin index being 0.559.

The silhouette coefficient was relatively large, indicating the satisfactory clustering effect of the model, the high similarity between countries within the divided communities, and the presence of significant differences between communities. The Davies–Bouldin index of this hyperparameter group was 0.559, which is still within a reasonable range, indicating good separation between communities. In combination with the silhouette coefficient, it can be judged that the model can balance the closeness within a community against the separation between communities.

#### 4.2.2. Community Structure

On the basis of the hyperparameter group, this paper subjects the feature extraction results to dimensionality reduction and displays their distribution in low-dimensional spaces in a visual mode, as shown below.

(1) Community Numbers

The number of countries within each trade community in 2020 and 2023 reflects the evolving structure of the global RO/RO shipping network. Compared to 2020, the network in 2023 includes 21 additional countries. New additions in Africa, such as Kenya, Algeria, Mauritius, and Djibouti, span regions in North Africa, East Africa, and the Gulf of Aden. In Asia, countries like Vietnam and Cambodia are integrated as well. Based on Table 5, we conclude that C_0_, C_4_, and C_6_ are the most significant trade communities. These communities are not only the largest in terms of member countries but also encompass the highest number of hub ports, indicating their central roles in the RO/RO shipping network. It is also notable that C0 consistently remains the largest trade community, while C6 exhibits the fastest growth in terms of country additions and network expansion.

C_0_ has the largest number of countries, with 25, indicating that it is the dominant community in the RO/RO shipping network. C_4_ and C_6_ each have 19 countries, following closely behind, suggesting that these communities are of similar size and have a significant presence in the network. C_1_ (14 countries) and C_7_ (13 countries) are medium-sized communities, showing moderate connectivity. C_3_ and C_5_, with 11 countries each, are smaller in scale but still maintain a certain level of influence within the network. C_2_, with only 6 countries, is the smallest community, indicating weaker connectivity or higher concentration. Overall, the differences in community size highlight the varying levels of influence and importance of different communities within the global RO/RO shipping network.

(2) Community detection of the RO/RO shipping network based on the GIN model

**Figure 9:**
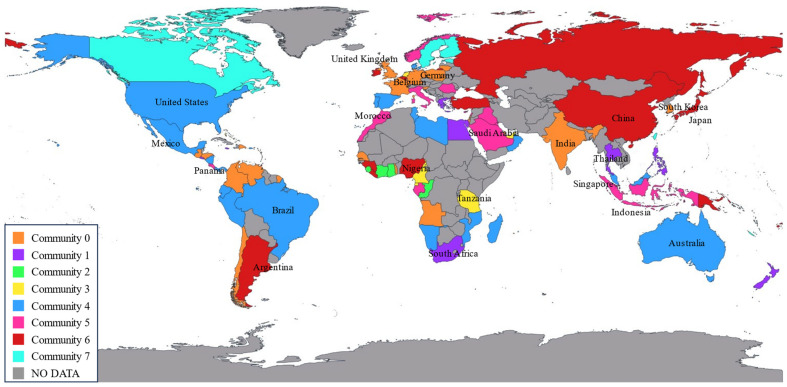
Visualization of community detection of country-level networks globally based on the GIN model (2020).

**Figure 10:**
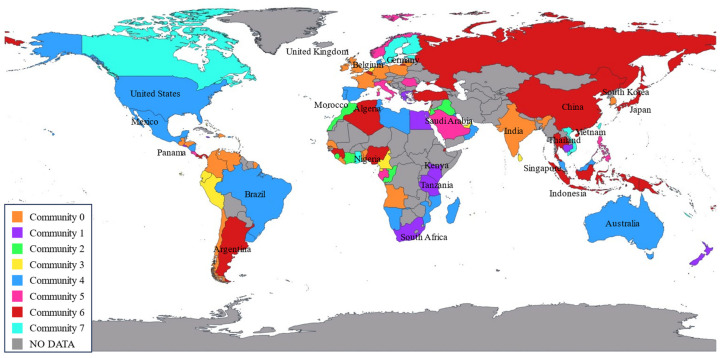
Visualization of community detection of country-level networks globally based on the GIN model (2023).

As shown in Figure 9 and Figure 10, the GIN-based community detection map illustrates the clear distribution of different communities within the global RO/RO shipping network. This pattern of distribution reveals the presence of distinct community structures in the global RO/RO shipping network, with each community representing a relatively independent region or group of countries, playing specific roles within the network. Compared to 2013, the global RO/RO shipping network in 2023 underwent changes, with countries in Africa and Southeast Asia emerging as new nodes within the network. This development strengthened cross-regional connectivity, facilitating trade flows and aligning with the projections outlined in the United Nations 2024 Global Trade Outlook [51]. Additionally, it enhanced the network’s resilience to economic fluctuations. By 2023, the number of trade communities increased, with Community 0, Community 4, and Community 6 emerging as the most influential clusters.

(3) The characteristics of the communities

**Figure 11:**
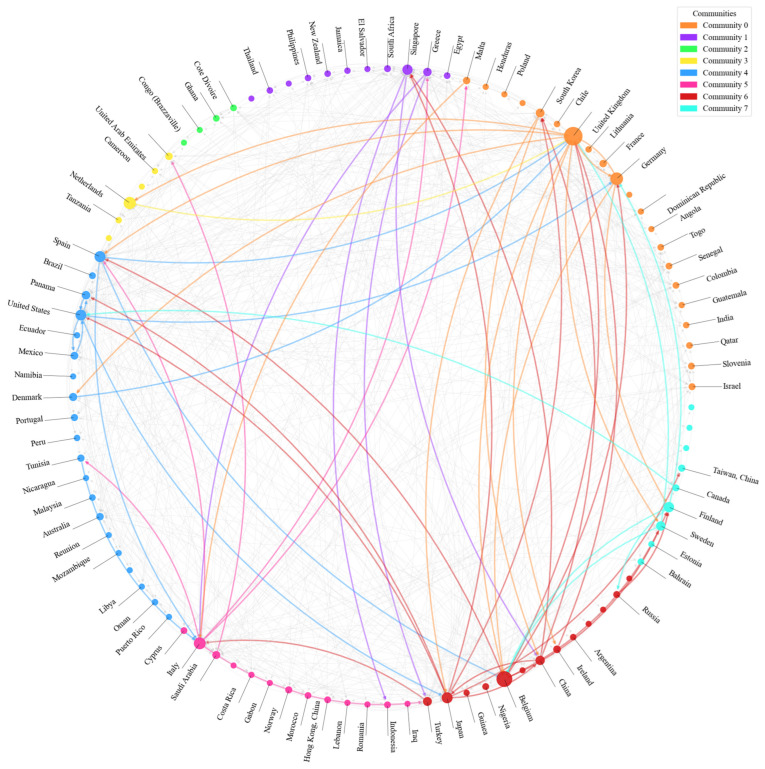
Network plot of the RO/RO shipping network with the communities (2020).

**Figure 12:**
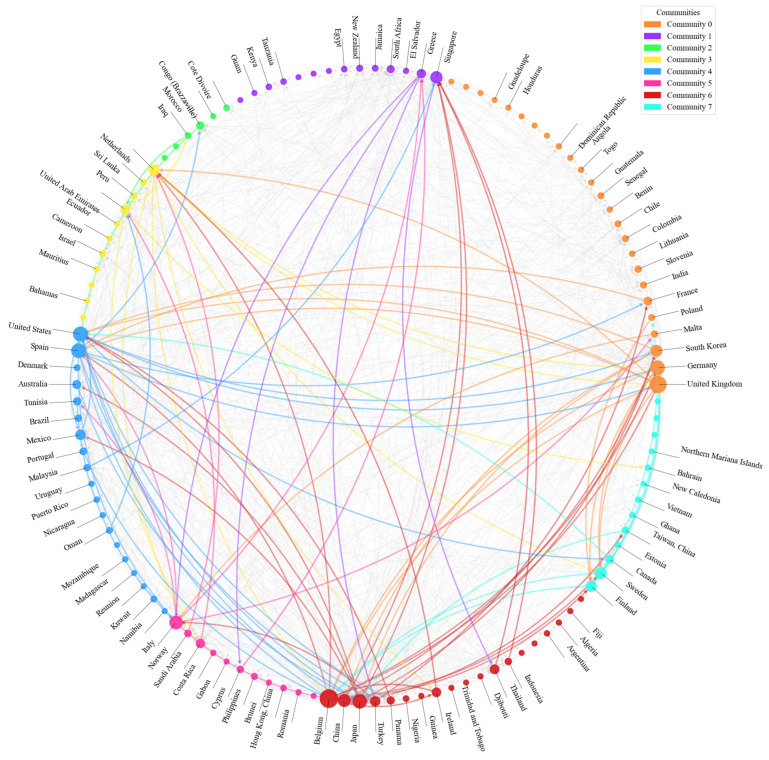
Network plot of the RO/RO shipping network with the communities (2023).

Based on the given community route data and the visual representation of the global RO/RO shipping network, the density of routes between communities is clearly illustrated, reflecting significant connections between different regions within the network, as detailed in Figure 11 and Figure 12. Here is an analysis of each community and its key countries.

As shown in Figure 12, Community 0 plays a central role in the global RO/RO shipping network, including key countries such as the United Kingdom, Germany, and France. Not only does Community 0 have strong internal connections (30 routes), but it also undertakes transport and transshipment functions with other important communities. There are 70 routes between Community 0 and Community 6 (which includes countries like Japan, China, and Belgium), highlighting the close cooperation between these European and Asian countries, particularly in vehicle exports and transshipment. Similarly, Community 0 has 70 routes with Community 4 (including the United States, Spain, and Australia), demonstrating high-frequency vehicle transport across the Atlantic, with major ports such as London and Hamburg supporting the transatlantic logistics network.

Community 1 (including countries like Greece, Singapore, and South Africa) handles some regional transshipment functions, particularly on the routes connecting Community 6 (44 routes) and Community 4 (42 routes). Singapore, as a key hub in Asia–Pacific, not only connects Asia but also acts as a transshipment point for African and European markets. These routes indicate Community 1’s crucial role in facilitating north-south trade globally, especially in connecting Europe and Africa.

Community 3, with key countries like the Netherlands, the UAE, and Israel, includes major transshipment ports like Rotterdam and Dubai, which handle significant transshipment activities. There are 36 routes between Community 3 and Community 6, particularly connecting Europe and the Middle East through ports in the Netherlands and Belgium. In addition, Community 3 has 35 routes with Community 5 (including Italy and Saudi Arabia), reflecting the tight logistics links between the Middle East and the Mediterranean region, driven by both transshipment and consumption demands.

Community 4 is a cross-regional trade community, including key countries like the United States, Spain, and Brazil. The 67 routes between Community 4 and Community 6 highlight the close logistics ties between the Atlantic and Pacific regions, particularly in global vehicle exports and heavy machinery transport. Major ports in Barcelona, Spain, and Los Angeles, United States, not only support intra-American logistics but also sustain intercontinental trade flows through these key connections.

Community 6, another key hub in the global shipping network, maintains not only close ties with Community 0 but also 67 routes with Community 4. Major ports in Japan (Yokohama) and China (Shanghai) serve as critical logistics bridges between Asia and Europe. Community 6 also maintains 29 routes with Community 7 (including Canada, Sweden, and Finland), showing its versatility in global logistics, not only serving major export markets but also maintaining stable logistics links with secondary markets.

Overall, Community 0 and Community 6 serve as core hubs in the global RO/RO shipping network, maintaining strong route connections with multiple communities and supporting global logistics operations. Community 1 and Community 4, through their regional hubs (such as ports in Singapore, South Africa, Spain, and the United States), effectively connect logistics networks between Europe, Asia, and the Americas. Community 3 and Community 5 are primarily responsible for the transportation of goods between the Middle East, the Mediterranean, and Europe, strengthening the flow of goods within these regions.

## 5. Discussion and Policy Implications

### 5.1. Discussion

The RO/RO shipping industry plays a pivotal role in facilitating global vehicle and machinery trade, and it continues to expand alongside the growth of international trade. As global automotive production increases, the importance of efficient RO/RO shipping becomes more prominent, contributing to the optimization of global supply chains. The development of the RO/RO sector supports the smooth flow of goods across continents, enabling faster and more efficient transport of vehicles and heavy machinery. Our study investigates the structure of the global RO/RO shipping network, providing a detailed analysis. Additionally, this research provides valuable insights for policymakers and industry stakeholders to better invest in the expansion of key transshipment hubs.

One of the most notable findings from the community detection analysis is the emergence of well-defined clusters of countries that are strongly linked through RO/RO shipping routes. The network exhibits significant modularity (0.403), which means that there are distinct groups of countries that tend to trade within their regions. For instance, Community 0, which includes the UK, France, Germany, India, and South Korea, controls approximately 39% of the global RO/RO shipping routes, with a large concentration of trade within Europe and extending into Asia. The existence of this cluster suggests that RO/RO trade in Europe has reached a considerable scale, with major European ports like London, Hamburg, and Rotterdam acting as central nodes in this network.

The modularity score also highlights the regionality of the RO/RO shipping network. For example, Community 6, which includes China, Japan, Belgium, Argentina, Turkey, and Thailand, handles 38% of the global routes. This community is notable for containing two of the world’s largest automobile exporters: China and Japan. These countries not only export vehicles to Europe and North America but also serve as important hubs for emerging markets in South America, such as Argentina. The growing importance of China as a vehicle exporter is reflected in the extensive network of routes connecting its ports, including Shanghai, Tianjin, and Shenzhen, with ports in Europe and beyond.

Another key community is Community 1, comprising Singapore, Egypt, South Africa, Guam, and New Zealand, which manages 23% of global routes. This community functions as a major transshipment hub for goods moving between Asia–Pacific, Africa, and the Middle East. Ports like Singapore, which are strategically located along major shipping routes, serve as critical points for the transshipment of vehicles, reducing the need for long-haul voyages and enhancing trade efficiency across regions.

The hierarchical coefficient of 0.575 observed in the network highlights the presence of a layered structure within the global RO/RO shipping network. Certain ports act as dominant nodes, not only controlling the flow of goods within their immediate region but also serving as gateways for other parts of the world. For instance, ports in the UK and Germany (Community 0) or China and Japan (Community 6) often function as critical nodes that facilitate the movement of goods between smaller ports in less connected regions. However, the hierarchical nature of the network also raises questions about its resilience. A highly hierarchical network tends to rely heavily on a few key hubs, which could become bottlenecks in the event of disruptions. For instance, if a major port like Singapore or Shanghai experiences delays or capacity issues, the ripple effects could extend throughout the global RO/RO shipping network, affecting the timely delivery of vehicles and machinery.

A comparative analysis of community distribution in the RO/RO shipping network between 2020 and 2023 reveals notable additions of new nodes and community shifts. Countries such as Vietnam, Djibouti, and other African nations saw increased engagement within the network, while nations including Indonesia, Israel, Tanzania, Thailand, Ghana, the Philippines, and Panama underwent community changes. The primary factors contributing to these shifts are as follows:

The increased RO/RO shipping volume, alongside the improvement in and recovery of network metrics, indicates the gradual restoration of the RO/RO shipping network in the wake of the pandemic. This observation aligns with insights from the International Chamber of Commerce (ICC) in the 2023 Global Supply Chain Risk Report [52], which highlighted how the pandemic exposed vulnerabilities in global supply chains, prompting companies to diversify their logistics channels to reduce reliance on single hub ports. Consequently, countries and companies are increasingly establishing cross-regional direct routes and secondary ports, thereby constructing a more resilient logistics network.

The African Development Bank (AfDB) has significantly increased infrastructure investments in East Africa and sub-Saharan Africa, helping these regions emerge as vital logistics hubs [53]. Ports such as Kenya’s Mombasa and Djibouti are playing increasingly important roles in facilitating trade within Africa as well as with the Middle East and Asia. Additionally, a World Bank report [54] highlights China’s involvement in supporting Africa’s construction of both port and inland transportation corridors. The report also points to a 15-year concession contract signed in Senegal, covering vehicle procurement for the public transport system, underscoring Africa’s commitment to enhancing port and vehicle import capacities. Research from the Hong Kong Trade Development Council also demonstrates that Southeast Asian countries, such as Vietnam and Cambodia, are rapidly becoming global manufacturing centers [55]. Trade ties between these nations and East Asian countries like China and Japan have been strengthening, further promoting the development of regional RO/RO shipping routes.

The study’s results also underscore the network’s complexity and sparsity. While certain regions, particularly, Europe and Asia, are well connected, other regions, such as Africa and South America, exhibit lower connectivity. The sparse connections in these regions suggest inefficiencies that could be addressed through targeted investments in port infrastructure and logistics networks. Additionally, the uneven distribution of routes means that some countries, particularly those in Communities 2, 5, and 7, are more reliant on a few central hubs for trade, which can limit their trade flexibility.

As automobile production and export capacities in countries like China, Thailand, and Argentina continue to grow, the network will likely experience shifts in trade patterns. For example, the expansion of Chinese automobile manufacturing plants and increased vehicle exports are expected to create new trade routes, particularly between Asia and South America. These changes will not only enhance China’s position within the global RO/RO shipping network but also have a profound impact on the overall connectivity of the network.

Moreover, as South Africa and other countries develop into key transshipment hubs, there will be increased opportunities for regions like Africa and the Middle East to integrate more fully into the global network. This integration will be crucial for facilitating trade between historically underdeveloped regions and more established markets in Europe, Asia, and North America.

### 5.2. Policy Implications

Based on the findings of this study, several policy recommendations can be made to improve the efficiency, resilience, and sustainability of the global RO/RO shipping network. These policy implications aim to address the challenges of network sparsity, hierarchical bottlenecks, and the need for increased regional connectivity.

**Enhancing Port Infrastructure in Key Communities**. The development of regional core hubs, especially in communities like Community 6, which includes key automobile exporters such as China and Japan, should be prioritized. By upgrading ports in these countries to larger and more efficient transshipment hubs, the flow of automobiles between Asia, Europe, and South America can be strengthened, enhancing regional logistics efficiency, reducing bottlenecks in export processes, and achieving the goal of lowering logistics costs. Meanwhile, to address health and safety challenges, the automation of RO/RO terminals should be prioritized. Governments can support this transition by offering tax incentives, streamlining customs procedures, and facilitating the adoption of automated loading and unloading equipment, remote operation technologies, and intelligent monitoring systems, thereby increasing RO/RO terminal transportation efficiency and reducing operational costs.

**Encouraging Trade Route Diversification**. Policymakers should focus on diversifying the trade routes and logistics nodes within key communities to mitigate trade risks and enhance supply chain resilience. By providing incentives such as subsidies and tax benefits, shipping companies can be encouraged to develop new cross-regional routes [56], reducing reliance on a limited number of hub ports. For example, extending trade routes from Community 0 to Community 7 would help reduce over-reliance on a few key regions and increase flexibility in the event of trade disruptions. Moreover, strengthening the routes between Community 4 (comprising the US, Spain, and Brazil) and Community 5 (including Italy and Saudi Arabia) would facilitate new trade partnerships. Similarly, intra-community short-sea RO/RO routes have demonstrated lower costs compared to traditional loading and unloading methods, further highlighting the critical role of RO/RO transport within the maritime supply chain [57]. Therefore, policymakers should implement practical pathways to lower vessel transportation costs within communities, promoting sustainable regional development and resource balance.

**Strengthening Connectivity in Underdeveloped Regions.** The study highlights the lower connectivity in regions like Africa and South America (Community 2 and Community 5), which limits these regions’ ability to fully integrate into the global RO/RO shipping network. Policymakers should prioritize investments in port infrastructure and logistics networks in these regions to strengthen their connectivity with key global markets. Compared to container terminals, RO/RO terminals require less construction investment and have shorter construction cycles, as they do not require extensive investment in loading and unloading equipment [58]. Additionally, they are well suited for multimodal transport, making them ideal for preliminary port terminal development in countries within Communities 2 and 5. Public–private partnerships could be established to fund the development of modern, high-capacity ports in these regions, ensuring that they can handle larger volumes of RO/RO vessels and support increased trade. For instance, ports in Argentina and Brazil could be upgraded to serve as major gateways for trade between South America and other regions, while ports in North Africa and the Middle East could be enhanced to facilitate trade between Europe, Africa, and Asia.

**Reducing Trade Risks Through Network Resilience**. The hierarchical structure of the RO/RO shipping network, with its reliance on a few key hubs, creates vulnerabilities in the event of disruptions. To reduce these risks, policymakers should focus on increasing the resilience of the network by investing in backup infrastructure and contingency plans for key ports. This could include establishing secondary transshipment hubs in less congested ports or creating redundancies in port infrastructure to handle surges in demand or unexpected delays. Additionally, regional cooperation between countries could help develop contingency plans for major disruptions in global trade routes. By fostering stronger ties between countries in the same community, the global RO/RO shipping network can become more adaptable and resilient to future challenges.

## 6. Conclusions

This paper focuses on the community detection of the RO/RO shipping network, and simulates their structural features and dynamic changes in combination with the background of China’s automobile export. Existing studies often examine the features of RO/RO vessels from the perspectives of energy technology and emissions, and focus on the behavior or facilities of a single RO/RO vessel as the research object, making it impossible to discuss the behavior of RO/RO shipping between countries from a macro level. For this reason, existing studies cannot provide decision support for the efficient and sustainable development of the RO/RO shipping network. The RO/RO shipping network constructed and used in this paper can fully reflect the operations of RO/RO vessels at national and port levels, offering good data support for a multi-scale depiction of RO/RO vessels. In this paper, we adopt a set of complex network indicators and methods to construct a RO/RO shipping network from the perspective of transport using the AIS data on RO/RO vessels collected from 2020 to 2023. Our conclusions are as follows:

In the analysis of the RO/RO shipping network, we quantitatively assess RO/RO shipping network using network indicators such as complexity, sparsity, homogeneity, modularity, and hierarchy. The modularity score of 0.403 signifies clear community structures, while the hierarchical coefficient of 0.575 reflects a certain degree of hierarchy within the network. The RO/RO shipping network exhibits a high level of complexity, with a graph density of 0.105, a parameter that remained relatively stable from 2020 to 2023, with an average value of 0.11. The average degree increased by 7% from 4.224, indicating a rise in the average number of connections per country, which suggests that the network maintains its sparsity while demonstrating good connectivity. Modularity decreased by 6.5%, from 0.431 in 2023 to 0.403, while the hierarchy coefficient rose to 0.575. This indicates that, post-pandemic, the routes within communities became more concentrated, signaling a maturation of the RO/RO shipping network.

In the feature extraction and community detection of RO/RO vessels, we use an automatic network feature extraction method. Experiments reveal that after parameter optimization, the model obtained a silhouette coefficient of 0.548 and a Davies–Bouldin index of 0.559. In comparison, between 2020 and 2023, the changes in the silhouette coefficient and the Davies–Bouldin index were small. GINs can extract the features of the RO/RO shipping network in a desirable manner and provide interpretable community detection results based on a clustering algorithm.

From 2020 to 2023, the number of nations participating in the Ro/Ro shipping network increased by 22%, mainly concentrated in African countries. This expansion underscores the growing integration of African nations into the global RO/RO shipping network, driven by strategic investments in infrastructure and enhanced connectivity across the region. Analysis of the RO/RO shipping network using complex network metrics over the four-year period highlights a notable post-pandemic restructuring that led to improvements in network robustness, efficiency, and resilience. Key metrics such as the average clustering coefficient, average degree, and hierarchy coefficient showed upward trends, reflecting strengthened connections. These structural enhancements suggest that the RO/RO shipping network not only adapted but also evolved to support diversified trade routes and a wider array of global stakeholders.

The RO/RO shipping network exhibits a well-defined community structure with significant route coverage across key communities. The community that includes the UK, France, Germany, India, and South Korea handles 39% of global RO/RO shipping routes, covering approximately 300 routes, primarily within Europe and connecting to Asia. Another community comprising Singapore, Egypt, South Africa, Guam, and New Zealand manages 23% of global routes, with around 180 routes, serving as a transshipment hub for Asia–Pacific, Africa, and the Middle East. Meanwhile, the community with China, Argentina, Belgium, Japan, Turkey, and Thailand controls 38% of global routes, covering 300 routes and connecting major automobile exporters in Asia and Europe with emerging markets in South America.

This paper not only enriches the application of GNNs in shipping network analysis in theory but also provides valuable references for shipping logistics planning and decision-making. Future studies can further explore the effects of more dynamic factors on shipping networks and how to use more complex GNN models to improve the precision of community detection analysis.

## Figures and Tables

**Table 1:** Statistics of global RO/RO vessel entry and exit records from 2020 to 2023.

	2020	2021	2022	2023
Number of records	27,257	28,764	31,663	33,470
Number of ports	473	488	502	537
Number of routes	675	710	731	856
Number of nations	97	109	112	118

**Table 2:** The formula for network analysis metrics.

	Metrics	Formulas	Meaning of Variable
Complexity	Average clustering coefficient	$C=\frac{1}{\left|V\right|}\sum_{i\in V}C_{i},C_{i}=\frac{2e_{i}}{k_{i}\left(k_{i}-1\right)}$	$\left|V\right|$ is the total number of nodes in the graph. $C_{i}$ is the clustering coefficient of node *i*. $e_{i}\;$ is the number of edges between the neighbors of node *i*. $k_{i}\;$ is the degree of node *i*.
	Graph entropy	$H\left(G\right)=-\sum_{i=1}^{n}p\left(i\right)\log p\left(i\right)$	$p\left(i\right)$ is the proportion of nodes in the *ith* category. n is number of different categories.
	Spectral radius	$\rho \left(A\right)=\max\{\left|\lambda_{1}\right|,\left|\lambda_{2}\right|,\ldots ,\left|\lambda_{n}\right|\}$	$\lambda_{1},\lambda_{2},\ldots ,\lambda_{n}$ is the eigenvalue of the adjacency matrix A.
Sparsity	Graph density	$D=\frac{\left|E\right|}{\left|V\right|\times \left(\left|V\right|-1\right)}$	$\left|E\right|$ is the total number of edges in the graph. $\left|V\right|$ is the total number of nodes in the graph.
	Average degree	$k=\frac{\left|E\right|}{\left|V\right|}$	Same as graph density.
Homogeneity	Structural homogeneity	$H=1-\frac{\sigma \left(k\right)}{\bar{k}}$	$\sigma \left(k\right)$ is the standard deviation of node degrees. $\bar{k}$ is the average degree of the nodes.
Modularity	Modularity	$Q=\frac{1}{m}\sum_{i,j}\left[A_{ij}-\frac{k_{i}^{\mathrm{in}}k_{j}^{\mathrm{out}}}{m}\right]\delta \left(c_{i},c_{j}\right)$	$A_{ij}$ is the adjacency matrix element. $k_{i}^{\mathrm{in}}$ is the in-degree of node *i*. $k_{j}^{\mathrm{out}}$ is the out-degree of node *j*. $\delta \left(c_{i},c_{j}\right)$ is a delta function.
Hierarchy	Hierarchy coefficient	$HC=\frac{1}{\left|V\right|}\sum_{i=1}^{\left|V\right|}\frac{BC\left(i\right)}{\underset{j\in V}{\max}BC\left(j\right)}$	$BC\left(i\right)$ is the betweenness centrality of node *i*. $\underset{j\in V}{\max}BC\left(j\right)$ is the maximum betweenness centrality value among all nodes in the graph.
	Hierarchical modularity	$Q_{H}=\frac{1}{2\left|E\right|}\sum_{l=1}^{L}\sum_{i,j}\left[A_{ij}^{l}-\frac{k_{i}^{l}k_{j}^{l}}{2m_{l}}\right]\delta \left(c_{i}^{l},c_{j}^{l}\right)$	L is the total number of layers. $k_{i}^{l}$ is the degree of node *i* at layer *l*. $m_{l}$ is the total degree of layer *l*.
	Hierarchical entropy	$H=-\sum_{i=1}p_{i}\log\left(p_{i}\right)$	$p\left(i\right)$ is the proportion of nodes in the *ith* category.

**Table 3:** The clustering coefficient of the RO/RO shipping network from 2020 to 2023.

	2020	2021	2022	2023
Clustering coefficient	0.56	0.59	0.57	0.63

**Table 4:** Statistics of comprehensive evaluation indicators of the global RO/RO shipping network.

	Metrics	2020	2021	2022	2023	Random Graph
Complexity	Average clustering coefficient	0.561	0.592	0.564	0.656	0.204
	Graph entropy	4.244	4.271	4.284	4.538	4.908
	Spectral radius	2305	2675	2794	2033	14.237
Sparsity	Graph density	0.106	0.121	0.118	0.105	0.106
	Average degree	22.43	26.055	26.107	28.642	28.715
Homogeneity	Structural homogeneity	0.062	0.014	0.019	0.062	0.846
Modularity	Modularity	0.431	0.459	0.433	0.403	0.138
Hierarchy	Hierarchy coefficient	0.44	0.509	0.533	0.575	0.417
	Hierarchical modularity	0.863	0.919	0.865	1.087	1.206
	Hierarchical entropy	0.952	0.732	0.749	0.656	0.204

**Table 5:** Numbers of nations in different communities.

	C_0_	C_1_	C_2_	C_3_	C_4_	C_5_	C_6_	C_7_
2020	22	11	4	6	20	13	12	9
2023	25	14	6	11	19	11	19	13

## Data Availability

Data are contained within the article.

## References

[1] Paraskevadakis D., Ifeoluwa A. (2022). An industry-level analysis of the post-Brexit and post-COVID 19 Ro-Ro ferry market and critical maritime freight transport links between the UK and the EU. J. Shipp. Trade.

[2] Yoo J.Y. (2018). Global Logistics Trend Spillover Through Container and RoRo Global Logistics Trend Spillover Through Container and RoRo Shipping in North Europe Short Sea Shipping. Master’s Thesis.

[3] Zheng X.B., Kim Y.S., Shin Y.R. (2021). Cost effectiveness analysis in short sea shipping: Evidence from Northeast Asian routes. J. Mar. Sci. Eng..

[4] A UN Framework for the Immediate Socio-Economic Response to COVID-19. https://unsdg.un.org/resources/un-framework-immediate-socio-economic-response-covid-19.

[5] Grzelakowski A.S. (2022). The COVID 19 pandemic–challenges for maritime transport and global logistics supply chains. TransNav Int. J. Mar. Navig. Saf. Sea Transp..

[6] Jin L., Chen J., Chen Z., Sun X., Yu B. (2022). Impact of COVID-19 on China’s international liner shipping network based on AIS data. Transp. Policy.

[7] Michail N.A., Kostis D.M. (2020). Shipping markets in turmoil: An analysis of the COVID-19 outbreak and its implications. Transp. Res. Interdiscip. Perspect..

[8] Kurt I. (2023). Lessons learned from COVID-19 in terms of port operations: Evidence from Turkish ports. Res. Transp. Bus. Manag..

[9] Statement on the Fifteenth Meeting of the IHR (2005) Emergency Committee on the COVID-19 Pandemic. https://www.who.int//news/item/05-05-2023-statement-on-the-fifteenth-meeting-of-the-international-health-regulations-(2005)-emergency-committee-regarding-the-coronavirus-disease-(covid-19)-pandemic.

[10] Seddiek I.S., Ammar N.R. (2023). Technical and eco-environmental analysis of blue/green ammonia-fueled RO/RO ships. Transp. Res. Part D Transp. Environ..

[11] Jovanović I., Vladimir N., Perčić M., Koričan M. (2022). The feasibility of autonomous low-emission ro-ro passenger shipping in the Adriatic Sea. Ocean. Eng..

[12] Jia B., Rytter N.G.M., Reinhardt L.B., Haulot G., Billesø M.B. Estimating discharge time of cargo units—A case of Ro-Ro shipping. Proceedings of the 10th International Conference on Computational Logistics (ICCL 2019).

[13] Puisa R. (2021). Optimal stowage on Ro-Ro decks for efficiency and safety. J. Mar. Eng. Technol..

[14] Guerrero D., Letrouit L., Pais-Montes C. (2022). The container transport system during COVID-19: An analysis through the prism of complex networks. Transp. Policy.

[15] Qian B. (2021). Analysis of Southeast Asian Shipping Network Based on Complex Network. Int. Core J. Eng..

[16] Peng P., Poon J.P., Cheng S., Dong Y., Yang Y., Lu F. (2023). Container port influence: A spatial diffusion analysis of global transshipments. Marit. Policy Manag..

[17] Peng P., Yang Y., Lu F., Cheng S., Mou N., Yang R. (2018). Modelling the competitiveness of the ports along the Maritime Silk Road with big data. Transp. Res. Part A Policy Pract..

[18] Wang P., Hu Q., Xu Y., Mei Q., Wang F. (2021). Evaluation methods of port dominance: A critical review. Ocean. Coast. Manag..

[19] Peng P., Cheng S., Lu F. (2020). Characterizing the global liquefied petroleum gas trading community using mass vessel trajectory data. J. Clean. Prod..

[20] Peng P., Lu F., Cheng S., Yang Y. (2021). Mapping the global liquefied natural gas trade network: A perspective of maritime transportation. J. Clean. Prod..

[21] Zhang T., Xi D., Jiang W., Feng Y., Wang C., Hu X. (2024). Structural Characteristics and Evolution of a Weighted Sino-US Container Shipping Network. Chin. Geogr. Sci..

[22] Ge J., Zhang Q., Wan Z. (2022). Regional operating patterns of world container shipping network: A perspective from motif identification. Phys. A Stat. Mech. Its Appl..

[23] Ansorena I.L. (2018). Bilateral connectivity in the liner shipping network: An overview. World Rev. Intermodal Transp. Res..

[24] Calatayud A., Mangan J., Palacin R. (2017). Vulnerability of international freight flows to shipping network disruptions: A multiplex network perspective. Transp. Res. Part E Logist. Transp. Rev..

[25] Wu J., Lu J., Zhang L., Fan H. (2024). Spatial heterogeneity among different-sized port communities in directed-weighted global liner shipping network. J. Transp. Geogr..

[26] Zhang Q., Zeng Q. (2019). Analyzing the shipping network of the maritime silk road (MSR) based on a complex network. J. Coast. Res..

[27] Li Z., Li H., Zhang Q., Qi X. (2024). Data-driven research on the impact of COVID-19 on the global container shipping network. Ocean. Coast. Manag..

[28] Xu M., Zhu Y., Deng W., Shen Y., Li T. (2024). Assessing the efficiency and vulnerability of global liner shipping network. Glob. Netw..

[29] Wang L., Zhang N., Ye F., Lau Y.Y., Ducruet C. (2020). The complex network analysis of liner shipping networks: Lessons from the merger between COSCO and CSCL. Growth Chang..

[30] Peng P., Claramunt C., Cheng S., Yang Y., Lu F. (2023). A multi-layer modelling approach for mining versatile ports of a global maritime transportation network. Int. J. Digit. Earth.

[31] Semi-Supervised Classification with Graph Convolutional Networks. https://arxiv.org/abs/1609.02907.

[32] How Powerful Are Graph Neural Networks?. https://arxiv.org/abs/1810.00826.

[33] Velickovic P., Cucurull G., Casanova A., Romero A., Lio P., Bengio Y. (2017). Graph attention networks. Stat.

[34] Wen H., Lin Y., Mao X., Wu F., Zhao Y., Wang H., Wan H. Graph2route: A dynamic spatial-temporal graph neural network for pick-up and delivery route prediction. Proceedings of the 28th ACM SIGKDD conference on knowledge discovery and data mining.

[35] Diao C., Zhang D., Liang W., Li K.C., Hong Y., Gaudiot J.L. (2022). A novel spatial-temporal multi-scale alignment graph neural network security model for vehicles prediction. IEEE Trans. Intell. Transp. Syst..

[36] Wang X., Ma Y., Wang Y., Jin W., Wang X., Tang J., Yu J. Traffic flow prediction via spatial temporal graph neural network. Proceedings of the Web Conference 2020.

[37] Gammelli D., Yang K., Harrison J., Rodrigues F., Pereira F.C., Pavone M. Graph neural network reinforcement learning for autonomous mobility-on-demand systems. Proceedings of the 60th IEEE Conference on Decision and Control (CDC).

[38] Patel K., Bhatt C., Mazzeo P.L. (2022). Improved ship detection algorithm from satellite images using YOLOv7 and graph neural network. Algorithms.

[39] Altan D., Etemad M., Marijan D., Kholodna T. Discovering gateway ports in maritime using temporal graph neural network port classification. Proceedings of the 35th Canadian Conference on Artificial Intelligence.

[40] Xin R., Pan J., Yang F., Yan X., Ai B., Zhang Q. (2024). Graph deep learning recognition of port ship behavior patterns from a network approach. Ocean. Eng..

[41] Li L., Pan M., Liu Z., Sun H., Zhang R. (2024). Semi-dynamic spatial–temporal graph neural network for traffic state prediction in waterways. Ocean. Eng..

[42] Luo S., Zeng W. (2023). Vessel Trajectory Similarity Computation Based on Heterogeneous Graph Neural Network. J. Mar. Sci. Eng..

[43] Li Y., Li Z., Mei Q., Wang P., Hu W., Wang Z., Xie W., Yang Y., Chen Y. (2023). Research on multi-port ship traffic prediction method based on spatiotemporal graph neural networks. J. Mar. Sci. Eng..

[44] Zheng G., Chai W.K., Zhang J., Katos V. (2023). VDGCNeT: A novel network-wide Virtual Dynamic Graph Convolution Neural network and Transformer-based traffic prediction model. Knowl. Based Syst..

[45] Izadi S., Ahmadi M., Nikbazm R. (2022). Network traffic classification using convolutional neural network and ant-lion optimization. Comput. Electr. Eng..

[46] D’Angelo G., Palmieri F. (2021). Network traffic classification using deep convolutional recurrent autoencoder neural networks for spatial–temporal features extraction. J. Netw. Comput. Appl..

[47] Hevey D. (2018). Network analysis: A brief overview and tutorial. Health Psychol. Behav. Med..

[48] De Domenico M., Biamonte J. (2016). Spectral entropies as information-theoretic tools for complex network comparison. Phys. Rev. X.

[49] Franz M., Alberts S.C. (2015). Social network dynamics: The importance of distinguishing between heterogeneous and homogeneous changes. Behav. Ecol. Sociobiol..

[50] Javed M.A., Younis M.S., Latif S., Qadir J., Baig A. (2018). Community detection in networks: A multidisciplinary review. J. Netw. Comput. Appl..

[51] Global Trade Outlook and Statistics. https://www.wto.org/english/res_e/publications_e/trade_outlook24_e.htm.

[52] Global Supply Chain Risk Report 2023. https://www.wtwco.com/zh-tw/insights/2023/02/2023-global-supply-chain-risk-report.

[53] African Economic Outlook 2023. https://www.afdb.org/en/documents/african-economic-outlook-2023.

[54] China-World Bank Group Partnership Fund Progress Report. https://thedocs.worldbank.org/en/doc/3d9695e1d08f8e81fad7e70966976cb9-0060072023/original/CWPF-Progress-Report-FY17-FY22-Chinese.pdf.

[55] ASEAN Market Overview. https://research.hktdc.com/sc/article/Mzk5MzcxNjEz.

[56] Kimata J., Okamoto T., Takebayashi M. (2019). How to Establish West Japan-ASEAN RORO Ship Route?: Based on Network Analysis. J. East. Asia Soc. Transp. Stud..

[57] Krüger S., Marius S.M., Jahn C. (2022). Potential of container terminal operations for RoRo terminals. Changing Tides: The New Role of Resilience and Sustainability in Logistics and Supply Chain Management–Innovative Approaches for the Shift to a New Era, Proceedings of the Hamburg International Conference of Logistics (HICL), Hamburg, Germany, 21–23 September 2022.

[58] Santos T.A., Fonseca M.Â., Martins P., Soares C.G. (2022). Integrating short sea shipping with trans-European transport networks. J. Mar. Sci. Eng..

